# Comprehensive effects of N, P, K, and Zn formulated fertilization on growth, physiology, and seedling quality of *Carya illinoensis*

**DOI:** 10.3389/fpls.2026.1927567

**Published:** 2026-09-10

**Authors:** Zhaocheng Wang, Cheng Huang, Qingqing Wu, Changjian Qi, Aimin Wu, Yilin Ma, Xu Li, Xiang Ge, Wenyong Zhan, Longlong Li, Hua Liu

**Affiliations:** 1 School of Tourism Management, Huzhou Vocational and Technical College, Huzhou, China; 2 School of Forestry & Landscape Architecture, Anhui Agricultural University, Hefei, China; 3 Anhui Dechang Seedlings Co., Ltd., Lv’an, China; 4 Hubei Key Laboratory of Biologic Resources Protection and Utilization, Hubei Minzu University, Enshi, China; 5 Fuyang Yingquan District Forestry Technology Extension Center, Fuyang, China; 6 Huzhou Woye Ecological Agricultural Ltd., Huzhou, China; 7 South China Botanical Garden, Chinese Academy of Sciences, Guangzhou, China; 8 Fuyang Xinfeng Seed Industry Co., Ltd., Fuyang, China

**Keywords:** *Carya illinoensis*, formulated fertilization, nutrient interactions, physiological acclimation, seedling quality index

## Abstract

Optimizing fertilizer application is critical for balancing plant growth promotion, economic efficiency, and environmental sustainability. For *Carya illinoensis* (pecan), a high-value nut tree, balanced nutrient management of nitrogen (N), phosphorus (P), potassium (K), and zinc (Zn) is essential to enhance seedling vigor and establishment success. However, the optimal formulations of N, P, K, and Zn for maximizing seedling quality remain undetermined. This study employed an L9(3^4^) orthogonal design to evaluate the integrated effects of N, P, K, and Zn combinations on growth, physiological traits, and seedling quality index (SQI) of pecan seedlings. The results demonstrated that fertilization generally promoted growth and nutrient uptake, with responses varying across parameters. Specifically, the N1P3K3Zn3 treatment (low N, high P, high K, high Zn) achieved the greatest total biomass accumulation and SQI, while the N1P2K2Zn2 treatment (low N, medium P, medium K, medium Zn) maximized ground diameter growth. Physiological analyses revealed that fertilization enhanced leaf chlorophyll content and non-structural carbohydrate accumulation during the active growth phase (June-August). Furthermore, distinct formulations differentially modulated stress responses; for instance, N2P1K2Zn3 minimized malondialdehyde content, while N2P2K3Zn1 optimized leaf membrane stability. Principal component analysis of 14 integrated indicators identified N1P2K2Zn2 as the treatment yielding the best overall seedling performance. These findings underscore that nutrient interactions, rather than single-element effects, drive seedling quality in pecan. The study provides a quantitative basis for formulating balanced N-P-K-Zn fertilizers to cultivate high-quality pecan seedlings, thereby supporting sustainable nursery management and afforestation practices. Future work should validate these formulations in long-term field trials and assess their impacts on orchard productivity and soil health.

## 1 Introduction

Formulated fertilization, which tailors the proportions of nutrient elements based on plant requirements and soil fertility, is a critical strategy for enhancing plant growth and nutrient use efficiency (Burducea et al., 2018; De la Portilla et al., 2020; Gavric et al., 2021). In forestry and horticulture, the rational application of macronutrients, nitrogen (N), phosphorus (P), and potassium (K), alongside essential micronutrients such as zinc (Zn), is fundamental for seedling cultivation (Wang et al., 2019; Cambareri et al., 2023a; Li et al., 2024a). While the individual roles of these elements are well-established, N in protein synthesis and vegetative growth, P in energy transfer and root development, K in osmotic regulation and stress resistance, and Zn in hormone regulation and enzymatic activity, their interactive effects are complex and often species-specific (Kormawa et al., 2003; Lamb, 2003; Hu et al., 2007; Faruque-As-Sunny et al., 2018). Previous studies on various tree species have demonstrated that specific N, P, and K formulations significantly affect growth and physiological traits (Cao et al., 2013; Cruz et al., 2019; Lazarevic et al., 2020; Attia et al., 2022). However, most existing research has focused on NPK combinations, often overlooking the synergistic or antagonistic interactions between micronutrients like Zn and macronutrients. This gap hinders the development of truly balanced fertilization regimes that maximize seedling quality.

*Carya illinoensis* (Wangenh.) K. Koch (Pecan) is a globally significant economic tree species valued for its high-quality nuts and timber (Wu et al., 2012; Zheng et al., 2015; Shi et al., 2018; Zhang et al., 2018). Introduced to China in the early 20th century, it has become a key species for afforestation and economic development in regions such as Anhui and Zhejiang (Zhao et al., 2007). The successful establishment of pecan plantations relies heavily on the cultivation of high-quality seedlings, which requires precise nutrient management. While previous studies have examined N, P, and K effects on pecan growth (Luo et al., 2019), limited research has explored the combined effects of N, P, K, and Zn. Specifically, it remains unclear how Zn interacts with NPK to influence nutrient uptake, carbohydrate metabolism, and stress physiology in pecan seedlings. Furthermore, excessive or imbalanced fertilization can lead to nutrient antagonism, reduced physiological resistance, and environmental pollution, highlighting the need for optimized formulations (Fu et al., 2021).

Traditionally, fertilizer optimization has relied on univariate approaches or simple multifactorial comparisons, which may fail to capture the complex, non-linear interactions among nutrients (Gallardo et al., 2021). Recent advances in precision agriculture suggest that integrating nutrient diagnostic systems with machine-learning approaches can substantially improve fertilizer recommendations by identifying complex nutrient interactions and site-specific nutrient constraints (Xie et al., 2023). For instance, Rymbai et al. (2026) demonstrated that machine learning tools could effectively diagnose soil nutrient constraints in citrus orchards, highlighting the potential of data-driven methods in nutrient management (Xie et al., 2023). Similarly, the Diagnosis and Recommendation Integrated System (DRIS) has proven effective in identifying yield-limiting nutrients and optimizing fertilizer recommendations in fruit crops (Zhang et al., 2023). Inspired by these advances, this study employs an orthogonal experimental design (L9 (3^4^)) combined with multivariate statistical analysis and machine learning (Random Forest) to investigate the effects of N, P, K, and Zn combinations on pecan seedlings. This approach allows for a comprehensive evaluation of nutrient interactions and the identification of key factors driving seedling quality, offering a more robust scientific basis than conventional single-factor experiments.

The novelty of this study lies in three aspects: (1) it establishes a comprehensive evaluation system for pecan seedling quality that integrates growth, nutrient uptake, and physiological resistance under NPKZn interactions; (2) it employs an orthogonal design and Random Forest analysis to quantify the contribution of each nutrient element and identify the optimal formulation, addressing the limitations of previous single-factor or NPK-only studies; (3) it provides a theoretical basis for precise fertilization regimes for *C. illinoensis* nursery management. We hypothesized that balanced N, P, K, and Zn combinations would enhance seedling growth and quality by improving nutrient uptake and physiological stability, whereas unbalanced formulations would induce stress and reduce biomass accumulation. The findings aim to contribute to sustainable afforestation practices and efficient nutrient management for pecan cultivation.

## 2 Materials and methods

### 2.1 Study area

The experimental site is located at Nutty Pie Agricultural Co., Ltd. (32°00’-39°36’ N, 114°52’-116°57’ E), Hefei City, Anhui Province. The site has a subtropical monsoon climate with an average annual precipitation of 918.1 mm, average annual evapotranspiration of 897 mm, an average frost-free period of 224 days per year, and an average annual air temperature of 15.0°C.

### 2.2 Test materials

The test materials were uniformly sized and robustly grown 1(1)-0 (rootstock 1 year old, grafted 1 year later, transplanted 0 times) ‘Mahan’ grafted seedlings (average seedling height 89.0 cm, average ground diameter 13.1 mm). Containers were 20 cm × 26 cm (diameter × height) non-woven pots, each filled with 10 kg of soil. The seedling substrate was a mixture of yellow heart soil, charcoal soil, and perlite (1:1:1), with a pH of 6.7 ± 0.24. The baseline soil nutrient contents were as follows: total N (0.85 ± 0.19) mg g^-1^, total P (0.65 ± 0.12) mg g^-1^, total K (5.15 ± 0.35) mg g^-1^, available P (9.51 ± 0.46) mg kg^-1^, available Zn (0.23 ± 0.03) mg kg^-1^, soil bulk density (1.41 ± 0.10) g cm^-3^, and field water holding capacity (139.95 ± 22.71) g kg^-1^. The fertilizers used were urea (46% N), calcium superphosphate (12% P_2_O_5_), zinc sulfate (99% ZnSO_4_), and potassium sulfate (52% K_2_O). The seedlings were moved to the greenhouse at the end of December 2020 and managed under standard nursery practices, with a 4-month growth acclimation period before the experiment.

### 2.3 Experimental design

An L9 (3^4^) orthogonal experimental design was employed, setting four factors, N, P, K, and Zn, each at three fertilization levels (**Table 1**). The experiment commenced in April 2021, comprising nine fertilization treatment groups plus a control (CK) treated with clear water, with six replicates per treatment, totaling 60 plants. Except for the fertilizer treatments, all seedlings were maintained under identical routine management conditions (Shen et al., 2017; Cambareri et al., 2023b; Wang et al., 2023; Xu et al., 2024). Fertilizer solutions were prepared by dissolving the respective fertilizer amounts in 500 mL of water and applied by pouring 5 cm from the main root of each seedling. Fertilization was performed three times: in spring (April), summer (June), and autumn (August). The specific fertilizer amounts and treatment combinations are detailed in **Tables 1**, **2**.

**Table 1:** Experimental factors and levels.

Levels	N (g plant^-1^)	P_2_O_5_ (g plant^-1^)	K_2_O (g plant^-1^)	ZnSO_4_ (g plant^-1^)
1	4 (8.69)	2 (16.66)	2 (3.84)	0.1 (0.1)
2	6 (13.04)	3 (25.00)	3 (5.76)	0.2 (0.2)
3	8 (17.39)	4 (33.33)	4 (7.69)	0.3 (0.3)

In brackets is the actual fertilizer application amount (g plant^-1^).

**Table 2:** Experimental factors and levels.

Treatments code	Test factors and levels				Treatments group
	N (g plant^-1^)	P_2_O_5_ (g plant^-1^)	K_2_O (g plant^-1^)	ZnSO_4_ (g plant^-1^)	
CK	0	0	0	0	CK
T1	4	2	2	0.1	N1P1K1Zn1
T2	4	3	3	0.2	N1P2K2Zn2
T3	4	4	4	0.3	N1P3K3Zn3
T4	6	2	3	0.3	N2P1K2Zn3
T5	6	3	4	0.1	N2P2K3Zn1
T6	6	4	2	0.2	N2P3K1Zn2
T7	8	2	4	0.2	N3P1K3Zn2
T8	8	3	2	0.3	N3P2K1Zn3
T9	8	4	3	0.1	N3P3K2Zn1

### 2.4 Measurements

#### 2.4.1 Plant growth indexes

Growth indicators were measured before the experiment (April) and at two-month intervals post-fertilization (June, August, and October). Seedling height was measured with a steel tape measure (accuracy 0.1 cm), and ground diameter was measured with electronic vernier calipers (accuracy 0.01 mm).

Before fertilization (April 2021), three seedlings were harvested to determine baseline biomass. At the final harvest (October 2021), three seedlings per plot were sampled for biomass measurement. Seedlings were washed with distilled water and separated into above-ground and underground parts. Fresh weights were recorded, after which samples were oven-dried at 105°C for 20 min (to kill enzymes) and then dried to constant weight at 70°C. Dry weights were measured using an electronic balance (accuracy 0.01 g) (Wu et al., 2022; Li et al., 2025).

The Seedling Quality Index (SQI, Equation 1) was calculated using the modified Dickson Quality Index formula (Wu et al., 2022; Li et al., 2025), as follows:

(1) $$\mathrm{SQI}=\frac{\text{Total Dry Weight }(\text{g})}{\frac{\text{Height }(\text{cm})}{\text{Diameter }(\text{mm})}+\frac{\text{Stem Dry Weight }(\text{g})}{\text{Root Dry Weight }(\text{g})}}$$

#### 2.4.2 Plant nutrients and resistance physiological indicators

Healthy, mature leaves (the 3rd to 5th fully expanded compound leaves from the middle to upper canopy) were collected two months after each fertilizer application (June, August, and October). Soluble sugar and soluble starch contents were determined by the anthrone colorimetric method; total nitrogen content was determined by the sodium salicylate method; total P content was determined by the sulfuric acid digestion-molybdenum antimony method; malondialdehyde (MDA) content was determined by the thiobarbituric acid (TBA) colorimetric method; root vigor was determined by the TTC (2,3,5-triphenyltetrazolium chloride) staining method; and relative electrical conductivity was determined using a conductivity meter (Bisun, 1998; Du et al., 2020; Liu et al., 2025; Nawaz et al., 2025; Li et al., 2026).

#### 2.4.3 Photosynthesis physiological parameters

In August 2021, on a sunny and windless day between 08:30 and 11:30, three seedlings were randomly selected from each treatment. Healthy, mature functional leaves were chosen to measure instantaneous photosynthetic parameters using a LI-6400XT Portable Photosynthesis System (LI-COR, Inc., Lincoln, NE, USA). The light intensity was set at 1200 μmol m^-2^ s^-1^. Net photosynthetic rate, stomatal conductance, intercellular CO_2_ concentration (Ci), transpiration rate, saturated vapor pressure deficit (VPD), and water use efficiency (WUE) were recorded (Wu et al., 2020; Wu et al., 2023; He et al., 2024; Wu et al., 2024).

#### 2.4.4 Plant chlorophyll

Total chlorophyll, chlorophyll a, chlorophyll b, and carotenoid contents were determined using the ethanol extraction method (Li et al., 2024b; Li et al., 2026). Leaf samples (0.1 g fresh weight) were soaked in 10 mL of 80% acetone in the dark for 48 h until the tissues turned white. The absorbance of the extract was measured at 470 nm, 646 nm, and 663 nm using a spectrophotometer (UV-1800, Shimadzu, Japan). Measurements were performed in June, August, and October to capture seasonal dynamics.

### 2.5 Data analysis

All data were analyzed using R software (version 4.1.3) and SPSS Statistics 21. Data normality and homogeneity of variance were tested prior to analysis. To fully utilize the orthogonal design, Range Analysis (R analysis) was first conducted to evaluate the main effects of each factor (N, P, K, Zn) on the measured indicators. The range value for each factor was calculated to determine the factor importance ranking, where a larger R value indicates a greater influence of the factor on the target trait. For repeated measurements (growth, chlorophyll, and non-structural carbohydrates sampled in June, August, and October), a Linear Mixed Model (LMM) was employed to account for the non-independence of observations, with “Treatment” and “Time” as fixed effects and “Plant ID” as a random effect. For single-timepoint measurements (final biomass, photosynthesis), one-way Analysis of Variance (ANOVA) followed by Tukey’s Honestly Significant Difference (HSD) test was used for multiple comparisons.

Principal Component Analysis (PCA) was performed on the standardized dataset (z-score normalization) to comprehensively evaluate treatment effects based on 14 integrated indicators. The number of principal components retained was determined by the Kaiser criterion (eigenvalue > 1) and the “elbow point” of the scree plot. Random Forest (RF) analysis was used to screen key factors affecting the Seedling Quality Index. The model was constructed using the randomForest package in R, with parameters set to n_tree_ = 500 and mtry defaulting to the square root of the number of predictor variables. Model accuracy and stability were assessed using Out-Of-Bag (OOB) error estimates and 10-fold cross-validation. Variable importance was ranked based on the percentage increase in Mean Squared Error (%IncMSE), and variables with significant importance (*p* < 0.05) were identified as key drivers. All graphs were plotted using the *ggplot2*, *vegan*, *dplyr*, and *ggcor* packages in R software (version 4.1.3).

## 3 Results

### 3.1 Nutritional growth and seedling quality of *C. illinoensis* under different fertilizer treatments

**Figure 1:**
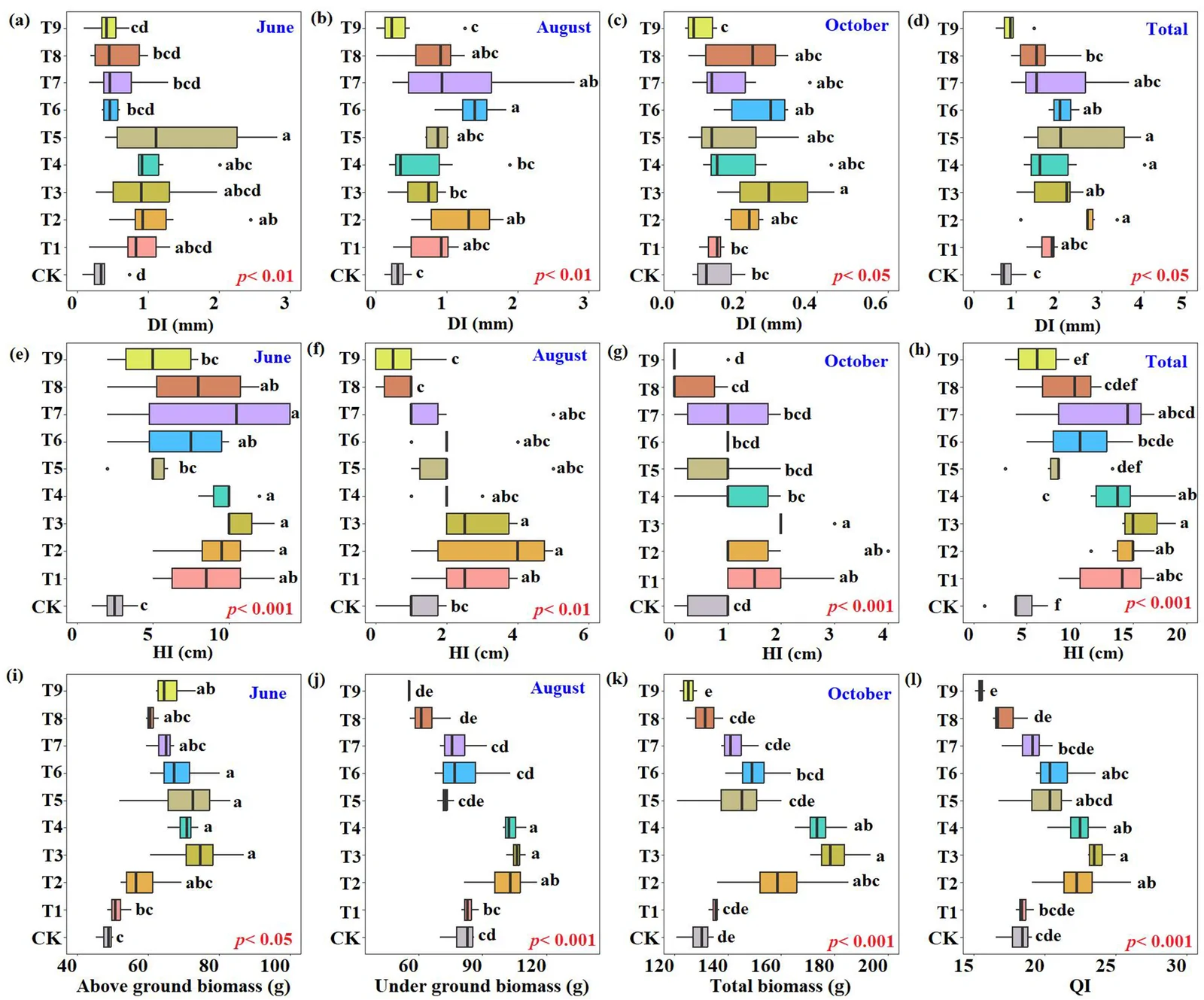
Nutritional growth and seedling quality of *C. illinoensis* seedlings under different fertilizer treatments. **(a)-(d)**, ground diameter (DI) in June, August, October, and cumulative growth (April-October); **(e)-(h)**, Seedling height (HI) in June, August, October, and cumulative growth (April-October); **(i)**, Above ground biomass; **(j)**, Underground biomass; **(k)**, Total biomass; **(l)**, Seedling quality index (QI). Different lowercase letters indicate significant differences between stands, and the LSD multiple test significance level is *p* < 0.05.

Range analysis (R analysis) of the orthogonal design results revealed the relative importance of each nutrient factor on the growth of *C. illinoensis* seedlings (**Table 3**). For seedling height and biomass, the range values followed the order N > Zn > P > K, indicating that N was the primary limiting factor, followed by Zn. For ground diameter, the order was N > P > Zn > K. The analysis identified the optimal factor combinations as N1P3K3Zn3 for maximizing height and biomass, and N1P2K2Zn2 for ground diameter (**Figure 1**).

**Table 3:** Range analysis of the height, ground diameter, and biomass of *Carya illinoensis* seedlings under different fertilization treatments.

Index	Factors	The effects of each level			Range	Optimum level	Effect order factors
		1	2	3			
Height/cm	N	14.39 ± 2.85a	10.56 ± 1.03b	8.89 ± 4.48b	5.50	N1	N>Zn>P>K
	P	12.94 ± 4.05a	10.27 ± 4.01a	10.61 ± 4.94a	2.67	P1	
	K	10.56 ± 3.84a	11.39 ± 4.58a	11.89 ± 4.96a	1.33	K3	
	Zn	8.94 ± 4.25b	12.11 ± 4.21a	12.78 ± 4.08a	3.84	Zn3	
Diameter/mm	N	2.07 ± 0.67a	2.18 ± 0.92a	1.45 ± 0.83ab	0.73	N2	N>P>Zn>K
	P	1.89 ± 0.86a	2.17 ± 0.97a	1.63 ± 0.68a	0.54	P2	
	K	1.78 ± 0.46a	1.82 ± 1.02a	2.10 ± 0.99a	0.32	K3	
	Zn	1.68 ± 0.96a	2.20 ± 0.79a	1.81 ± 0.78a	0.52	Zn2	
Biomass/g	N	158.35 ± 23.92a	155.79 ± 19.09a	129.73 ± 12.56b	28.62	N1	N>Zn>K>P
	P	150.70 ± 19.04a	142.33 ± 22.83ab	150.83 ± 26.86a	8.50	P3	
	K	136.59 ± 14.25ab	151.91 ± 27.01a	155.36 ± 22.38a	18.77	K3	
	Zn	133.32 ± 13.93bc	150.87 ± 16.09ab	159.67 ± 28.30a	26.35	Zn3	

Data are mean ± standard deviation, Different letters in the same treatment indicate significant difference at 0.05 level.

### 3.2 Effect of chlorophyll content and photosynthetic physiology of *C. illinoensis* seedlings under different fertilization treatments

**Figure 2:**
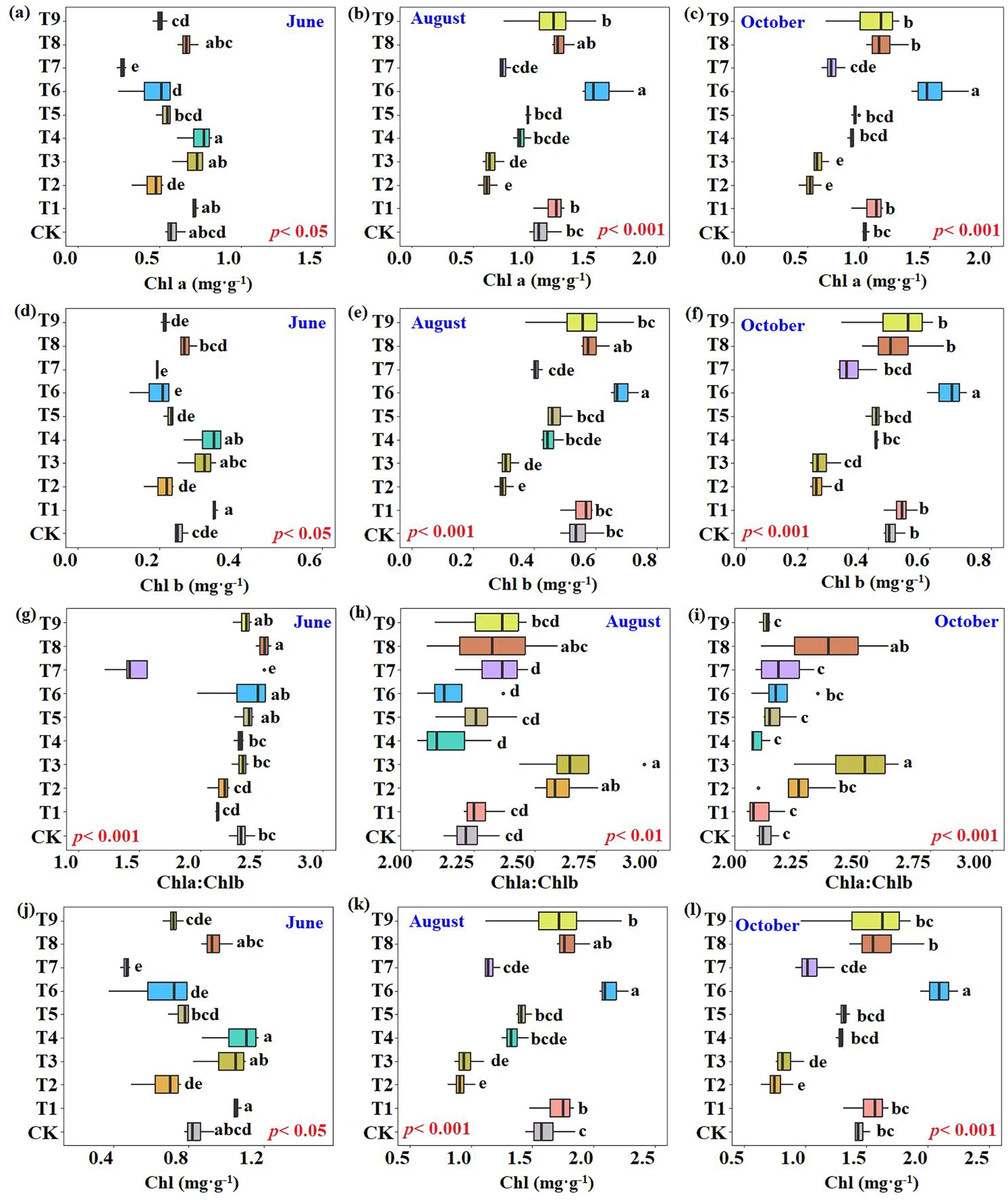
Chlorophyll content of *C. illinoensis* seedlings leaves under different fertilization treatments. **(a–c)**, Chlorophyll a content in June / August / October; **(d–f)**, Chlorophyll b content in June / August / October; (g–i), Chlorophyll a:b ratio in June / August / October; **(j–l)**, Total chlorophyll content in June / August / October. Different lowercase letters indicate significant differences between stands, and the LSD multiple test significance level is *p* < 0.05.

*C. illinoensis* seedlings had the highest total chlorophyll content of 1.06 mg g^-1^ under T4 treatment in spring (June), which was significantly (*p* < 0.05) 26.19% higher than CK (0.84 mg g^-1^) (**Figure 2**). The highest total chlorophyll content of T6 in summer (August) was 2.29 mg g^-1^, which was significantly higher (*p* < 0.05) than that of CK but significantly lower (*p* < 0.05) than that of CK under T2 and T3 treatments. T6 was still the highest (2.25 mg g^-1^), significantly higher (*p* < 0.05) than CK in autumn (October). Total chlorophyll increased from June to August and decreased from August to October in all treatments and CK, showing a consistent seasonal fluctuation pattern. The changes in chlorophyll a and chlorophyll b were the same as those of total chlorophyll. As far as Chla/Chlb was concerned, Chla/Chlb of thin-shelled pecan leaves under T3 treatment was the highest in August and October and significantly higher (*p* < 0.05) than CK.

**Figure 3:**
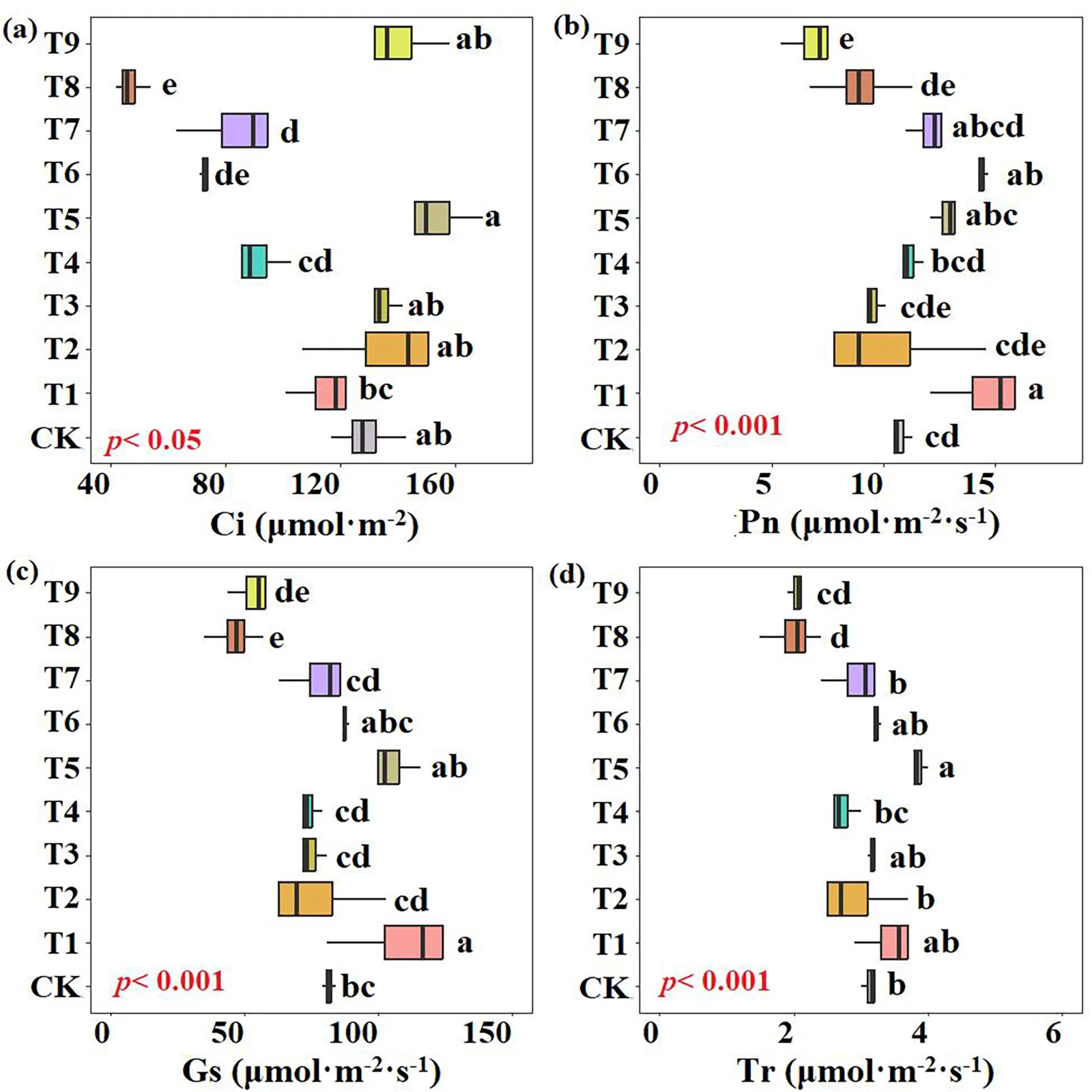
Photosynthetic physiological parameters of *C. illinoensis* seedlings leaves under different fertilization treatments. **(a)**, Intercellular CO2 concentration (Ci); **(b)**, Net photosynthetic rate (Pn); **(c)**, Stomatal conductance (Gs); **(d)**, Transpiration rate (Tr). Different lowercase letters indicate significant differences between stands, and the LSD multiple test significance level is *p* < 0.05.

*C. illinoensis* seedlings had the highest leaf Ci under T5 treatment compared to CK, which increased by 19.38% compared to CK, while T8 treatment had the lowest, which decreased by 63.57% compared to CK (**Figure 3**). Leaf Pn was the highest under T1 treatment, which increased by 35.84% compared to CK, followed by T6, which increased by 33.98% compared to CK, and the lowest Pn was found in T9, which decreased by 36.86% compared to CK. The stomatal conductance leaves were the highest and increased by 34.28% compared to CK; T8 had the lowest Gs and decreased by 42.86% compared to CK. Tr was the highest in tetrapods under T5 treatment, increased by 23.64% compared to CK, and was significantly related to CK (*p* < 0.05); T8 had the lowest transpiration rate and decreased by 36.10% compared to CK.

### 3.3 Effect of N and P contents of *C. illinoensis* seedlings leaves under different fertilization treatments

**Figure 4:**
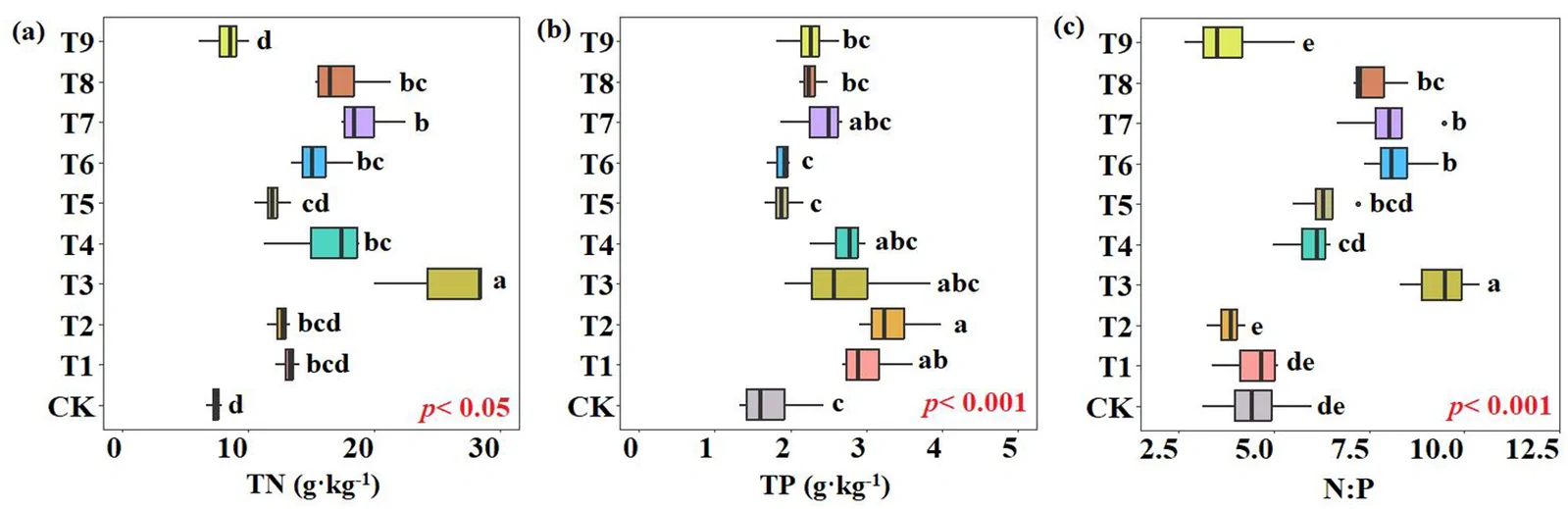
N and P contents of *C. illinoensis* seedlings leaves under different fertilization treatments. **(a)**, Total nitrogen (TN) content; **(b)**, Total phosphorus (TP) content; **(c)**, Nitrogen-to-phosphorus (N:P) ratio. Different lowercase letters indicate significant differences between stands, and the LSD multiple test significance level is *p* < 0.05.

*C. illinoensis* seedlings leaf N content was significantly higher (*p* < 0.05) than CK in all fertilization treatments except T9, with the highest leaf N content of 28.39 g kg^-1^ under T3 treatment, 282.61% higher than CK (**Figure 4**). Regarding leaf P content, only T2 and T3 treatments had significantly higher P content in leaves than CK (*p* < 0.05), while other treatments had no significant difference in leaf P content with CK. Leaf N:P ratio was highest in the T3 treatment, followed by T6, T7, and T8 treatments, all significantly higher than CK.

### 3.4 Effect of leaf non-structural carbohydrate content of *C. illinoensis* seedlings under different fertilization treatments

**Figure 5:**
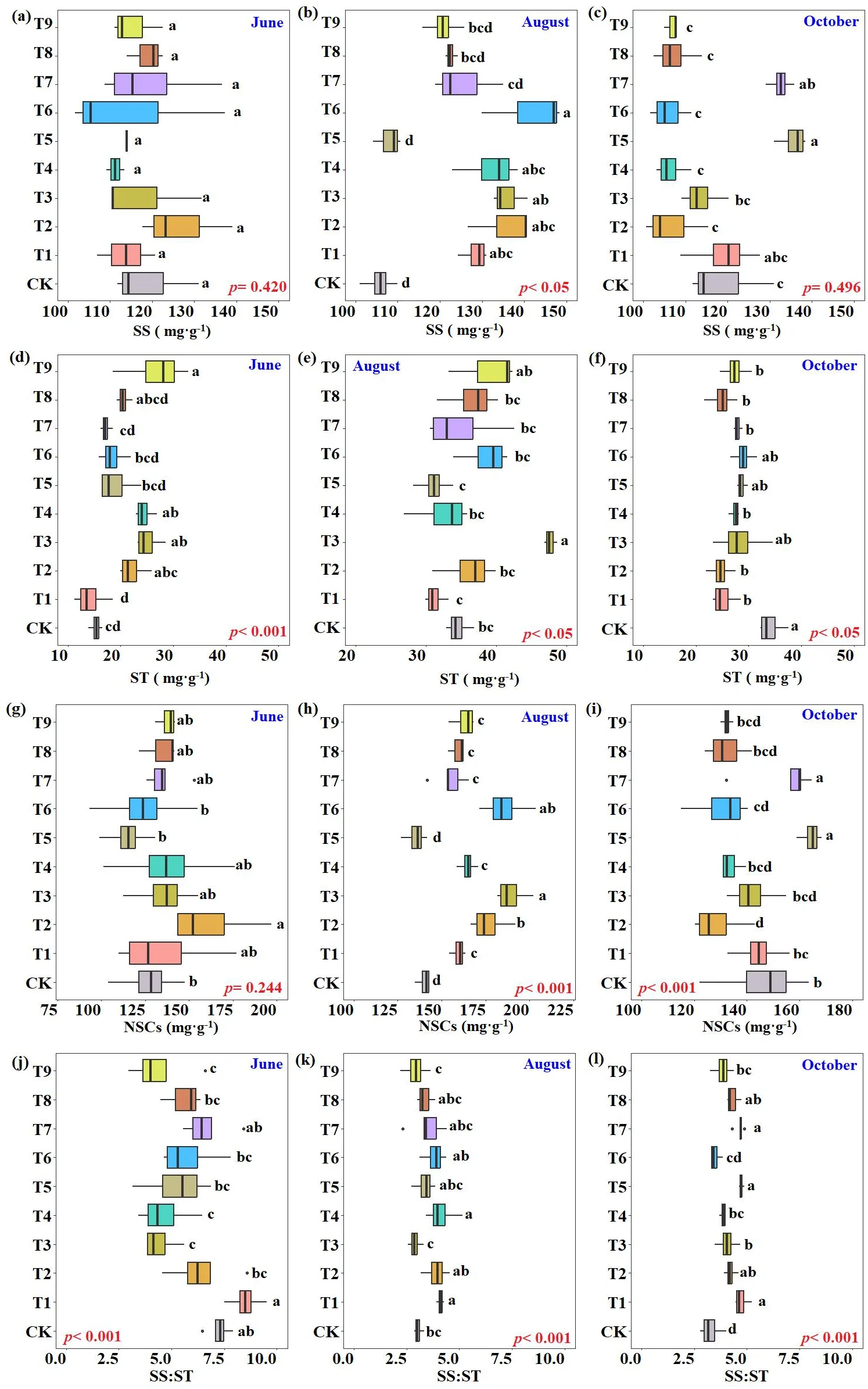
Non-structural carbohydrate content of *C. illinoensis* seedlings leaves under different fertilization treatments. **(a–c)**, Soluble sugar (SS) content in June / August / October; **(d–f)**, Soluble starch (ST) content in June / August / October; **(g–i)**, Total non-structural carbohydrate (NSC) content in June / August / October; **(j–l)**, SS:ST ratio in June / August / October. Different lowercase letters indicate significant differences between stands, and the LSD multiple test significance level is *p* < 0.05.

There was no significant difference in *C. illinoensis* seedlings’ leaf-soluble sugars under different fertilizer treatments in spring (June) (**Figure 5**). The highest leaf soluble sugar content of 147.05 mg g^-1^ was significantly (*p* < 0.05) higher than CK under T6 treatment in the summer (August). In autumn (October), the leaf-soluble sugar content was significantly higher (*p* < 0.05) than CK only under T5 and T7 treatments. Leaf-soluble starch content under T9 treatment was highest in spring (June) at 26.90 mg g^-1^, 75.24% higher than CK. In summer (August), soluble starch content was higher than that of CK in all treatment groups except T1 and T5, with treatment 3 being the highest at 48.68 mg g^-1^, which was significantly higher (*p* < 0.05) than that of CK; soluble starch content increased in all treatment groups compared with June. Among all the treatments, CK leaf-soluble starch content was the highest in October, followed by T6, T5, and T3. The analysis results showed that SS: ST was higher in all the periods under T1 treatment, while SS: ST was at a lower level in all the periods under T9 treatment, and the pattern of change of the leaf NSC content was the same as that of the leaf soluble sugar content.

### 3.5 Effect of root vigor and osmoregulatory substance content of *C. illinoensis* seedlings under different fertilizer treatments

**Figure 6:**
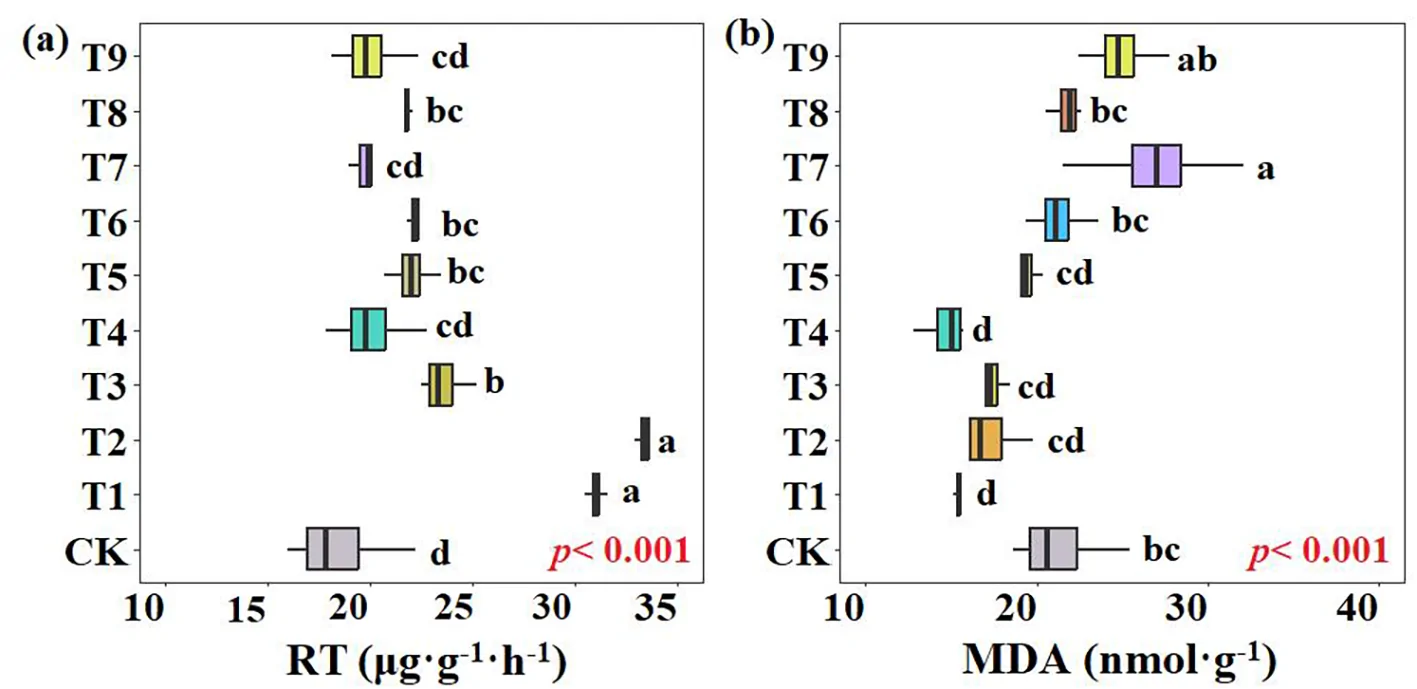
Root vigour and MDA content of *C. illinoensis* seedlings under different fertilizer treatments. **(a)**, Root vigor time (RT); **(b)**, Malondialdehyde (MDA) content. Different lowercase letters indicate significant differences between stands, and the LSD multiple test significance level is *p* < 0.05.

Root vigor was greater than CK (18.49 μg g^-1^ h^-1^) in all treatments, with T2 having the highest root vigor of 33.36 μg g^-1^ h^-1^, which was 80.42% higher than that of CK, followed by T1 (31.04 μg g^-1^ h^-1^), which was 67.87% higher than CK (**Figure 6**). All treatments’ root vigor was greater than CK’s (18.49 μg g^-1^ h^-1^). The malondialdehyde content of all fertilization treatments was less than that of CK (21.35 nmol g^-1^), except T7 (26.95 nmol g^-1^), T8 (21.77 nmol g^-1^), and treatment 9 (24.95 nmol g^-1^); among them, the malondialdehyde content of T4 was the lowest, 14.67 nmol g^-1^, which was 31.29% lower compared to CK; T7 had the lowest malondialdehyde content, 14.67 nmol g^-1^, and the malondialdehyde content of T7 was 31.29% higher than that of T7, followed by T1 (31.04 μg g^-1^ h^-1^, which was 67.1% higher than C The malondialdehyde content of treatment 4 was the lowest at 14.67 nmol g^-1^, which was 31.29% lower than that of CK, while that of T7 was the highest, which was 26.23% higher than that of CK.

### 3.6 Integrated trait analysis and key driving factors

**Figure 7:**
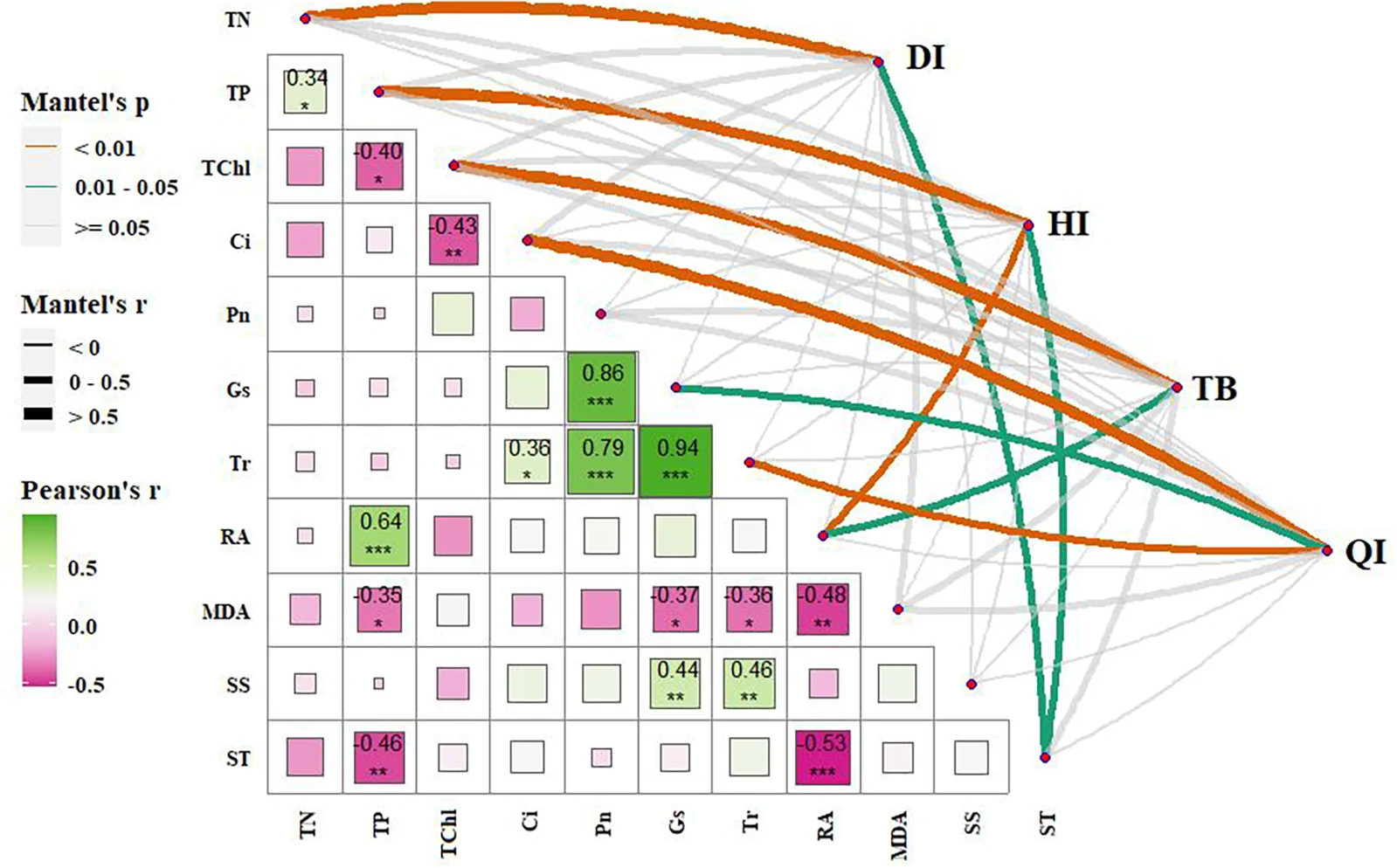
Correlation of *C. illinoensis* seedlings growth and quality with plant physiological indicators.

Mantel test analyzes showed that DI was highly significant and positively correlated with leaf ST content (*p* < 0.01), HI was significantly and positively correlated with leaf ST and SS: ST (*p* < 0.05), and TB was significantly and positively correlated with CHl and root MDA content was significantly and positively correlated with Pn, Gs, and MDA (*p* < 0.05) and highly and positively correlated with Tr (*p* < 0.01) (**Figure 7**).

**Figure 8:**
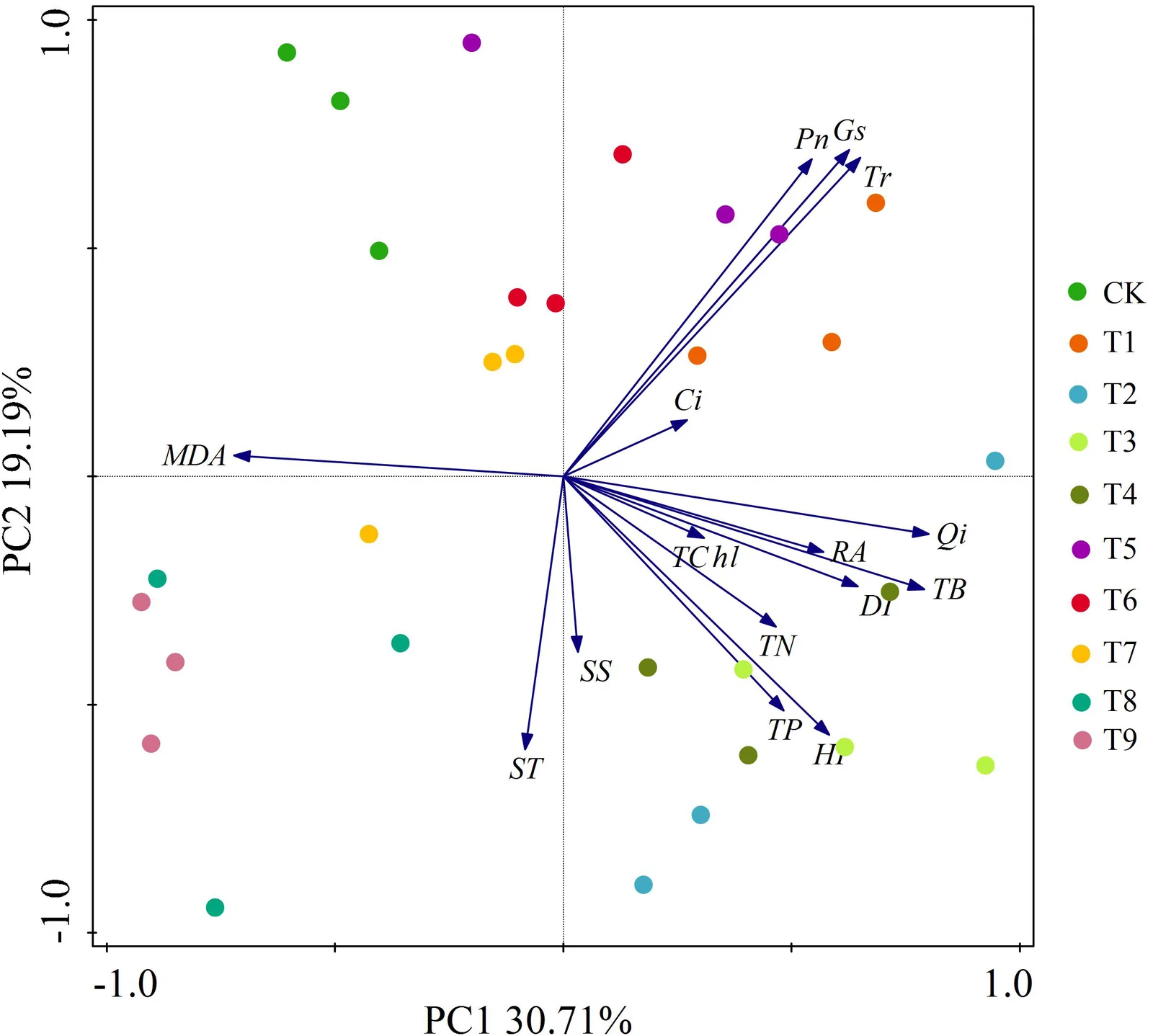
Principal component analysis of *C. illinoensis* seedlings growth and physiological characteristics.

Principal Component Analysis (PCA) based on 14 indicators explained 56.25% of the total variance in the first two components (**Figure 8**). PC1 (30.71%) was primarily loaded with biomass, SQI, and chlorophyll, representing the “growth-vigor” axis. PC2 (19.19%) was associated with oxidative stress indicators. The PCA biplot clearly separated the fertilization treatments from CK, with T2 and T3 positioned in the positive quadrant of PC1, indicating superior integrated performance.

**Figure 9:**
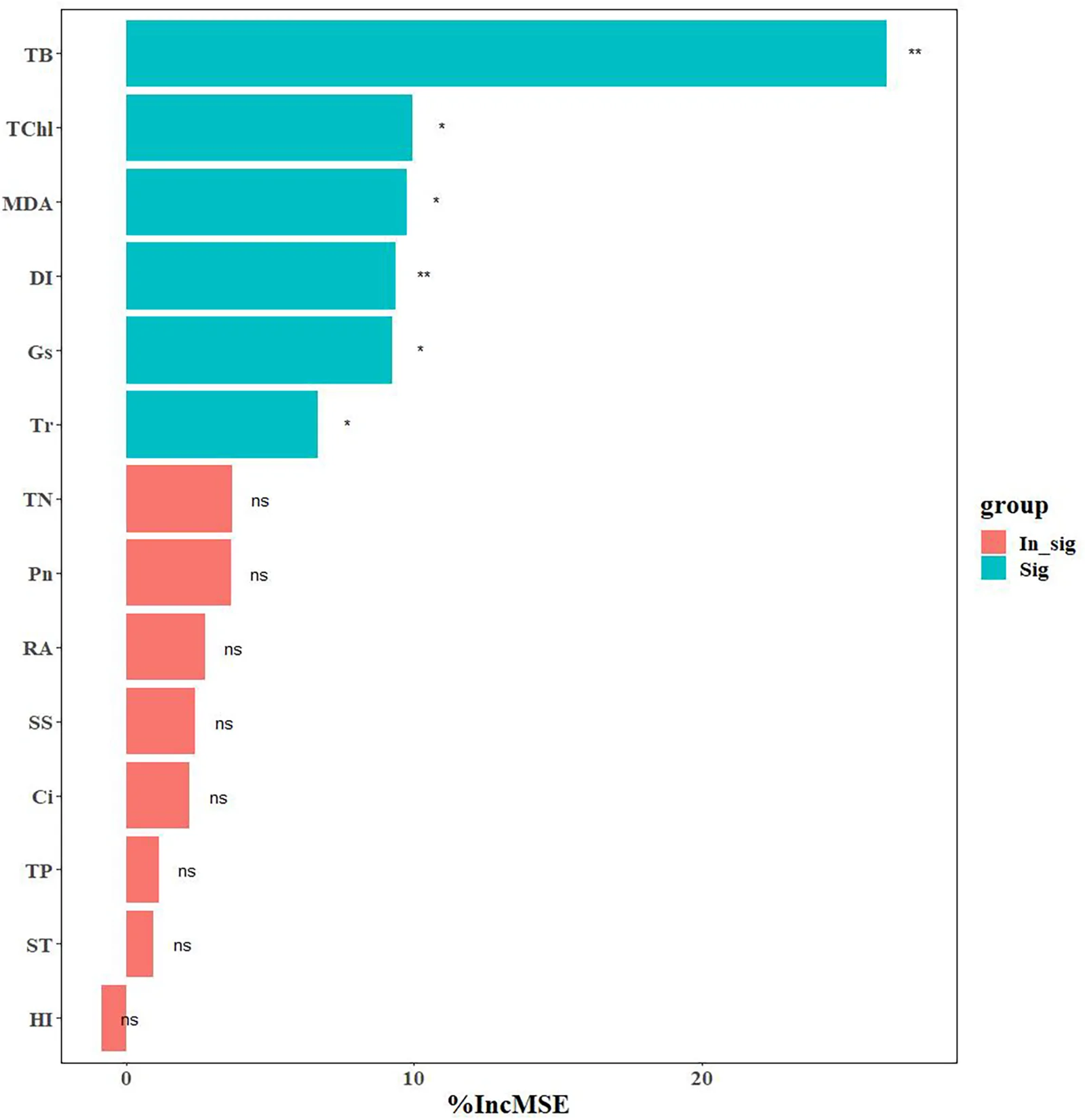
Influence of *C. illinoensis* seedlings growth and physiological characteristics on seedling quality.

Random Forest (RF) analysis identified Total Biomass (TB), MDA, and Stomatal Conductance as the most critical predictors of seedling quality (**Figure 9**). The model explained 85.4% of the variance in SQI (Out-of-Bag error rate < 0.05), with TB identified as the dominant factor (*p* < 0.01). Correlation analysis (**Figure 9**) further confirmed that SQI was positively correlated with biomass and chlorophyll but negatively correlated with MDA.

## 4 Discussion

### 4.1 Nutrient interactions drive growth variation: insights from orthogonal analysis

Consistent with the orthogonal range analysis, N emerged as the primary limiting factor for *C. illinoensis* growth, confirming its central role in protein synthesis and canopy development (Xie et al., 2014; Cheng et al., 2018; Stateras and Moustakas, 2018; Zhang et al., 2022). However, a key finding of this study was the critical, yet often overlooked, contribution of Zn. Ranked second only to N in influencing height growth, Zn demonstrated a significant synergistic effect with macronutrients. This aligns with the concept that balanced fertilization must account for micronutrient-macronutrient interactions (Fan et al., 2004; Zhu et al., 2012; Yang and Zheng, 2014; Xie et al., 2023). While moderate N levels (Level 1) maximized growth, higher N concentrations (Level 3, e.g., T7-T9) often resulted in reduced biomass accumulation. This “hump-shaped” response can be attributed to “luxury consumption” and ion antagonism; excessive N availability may inhibit the uptake of other essential cations like K and Ca, thereby disrupting metabolic balance and inducing physiological stress, as evidenced by elevated MDA content in high-N treatments (Wu et al., 2016; Wu et al., 2019; Wang et al., 2024). Similarly, the prominence of Zn in the orthogonal ranking highlights its non-replaceable role as a cofactor for auxin synthesis and carbonic anhydrase, which are crucial for cell elongation and photosynthesis (Zhu et al., 2012; Liao et al., 2018; Attia et al., 2022). These findings support the necessity of integrated nutrient management systems, such as the Diagnosis and Recommendation Integrated System (DRIS), which have proven effective in identifying yield-limiting nutrients and preventing the negative effects of over-fertilization in fruit crops (Yang and Zheng, 2014; Zhang et al., 2023).

### 4.2 Physiological cascade: from nutrient uptake to biomass accumulation

This study elucidates a coordinated physiological sequence linking fertilization to seedling quality. The improved SQI under balanced formulations (e.g., T2 and T3) was not an isolated event but the result of a cascade starting with nutrient uptake. The increased leaf N and P content directly facilitated the biosynthesis of photosynthetic pigments, as N is a core component of chlorophyll and P is essential for energy transfer (Li et al., 2009; Xia et al., 2014; Burducea et al., 2022; Wang et al., 2024). This was reflected in the significantly higher chlorophyll content observed in T3 and T6 during the active growth phase (August). Enhanced pigment levels subsequently drove higher net photosynthetic rates (Pn), leading to greater carbon assimilation. This assimilation was manifested as elevated soluble sugar and starch contents in leaves, representing the immediate products of photosynthesis and the substrate for growth (Wu et al., 2016; Liu et al., 2025). Finally, these non-structural carbohydrates were translocated and converted into structural biomass, resulting in greater height and diameter growth (Xue et al., 2020; Wang et al., 2024). This physiological trajectory, ranging from nutrient uptake to biomass accumulation, aligns with recent findings in dragon fruit propagation (Rymbai et al., 2025), where balanced nutrient supply and optimized management synergistically enhanced rooting and nutrient-use efficiency. Conversely, the decoupling of nutrient uptake and growth in high-N treatments (T7-T9), characterized by high leaf N but lower biomass and higher MDA, suggests a disruption in this metabolic chain, possibly due to nutrient imbalance inducing oxidative stress.

### 4.3 Zinc’s specific role in stress mitigation and carbon metabolism

Beyond its contribution to growth, Zn played a pivotal role in stress physiology. Treatments with appropriate Zn levels (e.g., T2, T4) exhibited significantly lower MDA content and higher root vigor compared to CK and high-N treatments. Zn is a structural component of superoxide dismutase (SOD), a key enzyme in scavenging reactive oxygen species (ROS) (Wu et al., 2016). The higher root vigor and lower lipid peroxidation under Zn-containing formulations indicate that Zn fortifies the antioxidant defense system, protecting root membranes during rapid nutrient absorption. Furthermore, the orthogonal analysis indicated that Zn was a major driver of soluble sugar content. This may be attributed to Zn’s role in regulating sucrose metabolism enzymes, ensuring efficient carbon partitioning between source leaves and sink roots (Zhu et al., 2012). This metabolic stability under Zn application is crucial for nursery stock, as it enhances transplanting resilience—a critical component of seedling quality often neglected by studies focusing solely on morphological parameters.

### 4.4 Trade-offs and multi-criteria optimization

A notable challenge in fertilization research is the divergence of optimal treatments for different variables; for instance, T3 maximized biomass but T2 maximized diameter. While high biomass (T3) indicates rapid growth, a robust ground diameter (T2) is often more correlated with survival and stability during field establishment (Wang et al., 2019). The PCA and SQI effectively reconciled these trade-offs. The superior SQI and positive PCA positioning of the N1P2K2Zn2 (T2) formulation, despite having lower total biomass than T3, suggest that seedling quality is a function of structural robustness and physiological balance rather than sheer size alone. The high N:P ratio in T3 suggests a potential P dilution effect due to rapid N-driven growth, whereas the balanced N:P ratio in T2 implies a more stable nutritional homeostasis. This reinforces the argument for multi-criteria decision analysis in nursery management, as optimizing for a single trait may compromise overall seedling vigor (Rymbai et al., 2024).

### 4.5 Implications for nursery management

The findings advocate for a shift from “blind” fertilization to precision nutrient management in *C. illinoensis* nurseries. The recommended formulation (N1P2K2Zn2) balances the high demand for N with adequate P and K, while incorporating Zn to mitigate stress and ensure metabolic efficiency. This approach not only improves the Seedling Quality Index but also enhances nutrient-use efficiency, reducing the environmental footprint associated with excessive N application (Fu et al., 2021). Future practices should integrate regular foliar nutrient diagnosis, potentially guided by DRIS norms established in this and similar studies (Zhang et al., 2023; Rymbai et al., 2024), to adjust fertilizer ratios dynamically according to seedling physiological status rather than static schedules. This data-driven approach aligns with modern precision agriculture trends and ensures the sustainable production of high-quality pecan planting stock.

## 5 Conclusions

This study employed an orthogonal design to evaluate the integrated effects of N, P, K, and Zn on *C. illinoensis* seedlings, revealing that nutrient interactions, rather than single-element effects, primarily drive seedling quality. The results demonstrated distinct physiological trade-offs among different formulations: the N1P3K3Zn3 formulation maximized total biomass accumulation and seedling height, whereas the N1P2K2Zn2 formulation resulted in the greatest ground diameter and the highest SQI. Multivariate analysis (PCA) identified N1P2K2Zn2 as the optimal formulation for overall seedling performance, as it balanced rapid growth with robust diameter development and improved physiological stability, as evidenced by lower MDA content and higher root vigor. Random Forest analysis highlighted that total biomass, antioxidant status, and stomatal conductance were key factors associated with SQI. These findings suggest that while high nutrient inputs can boost biomass, a balanced formulation (specifically low N, medium P, medium K, and medium Zn) is more effective for cultivating high-quality, resilient seedlings. This study provides a theoretical basis for precise fertilization in pecan nurseries, recommending N1P2K2Zn2 as a robust regime for general nursery management. Future research should focus on validating these formulations across diverse soil types and integrating nutrient diagnostic approaches (e.g., DRIS) to further refine site-specific recommendations for long-term orchard productivity.

## Data Availability

The raw data supporting the conclusions of this article will be made available by the authors, without undue reservation.
